# Exploring the neuroprotective role of GLP-1 agonists against Alzheimer's disease: Real-world evidence from a propensity-matched cohort

**DOI:** 10.1177/25424823251388650

**Published:** 2025-10-16

**Authors:** Majd A AbuAlrob, Adham Itbaisha, Yahya Kayed Abujwaid, Ayah Abulehia, Abdallah Hussein, Boulenouar Mesraoua

**Affiliations:** 1 Neurosciences Department, 36977 Hamad Medical Corporation, Doha, Qatar; 2 Faculty of Medicine, 61147 Al-Quds University, East Jerusalem, Palestine; 3 Department of Internal Medicine, 5948 Virtua Health, Camden, NJ, USA; 4 Weill Cornell Medicine–Qatar, Doha, Qatar

**Keywords:** Alzheimer's disease, diabetes, glucagon-like peptide-1 receptor agonists

## Abstract

**Background:**

Alzheimer's disease (AD) is a leading cause of dementia with societal and economic burdens. While recent therapies offer disease-modifying potential, concerns remain about efficacy and safety. Glucagon-like peptide-1 receptor agonists (GLP-1RAs), used in type 2 diabetes, show neuroprotective effects via reduced neuroinflammation and amyloid burden.

**Objective:**

To evaluate whether GLP-1RA use is associated with a reduced risk of incident dementia in adults aged ≥50 years using real-world data.

**Methods:**

We conducted a retrospective cohort study using the TriNetX platform, analyzing records from 142 healthcare organizations. Adults aged ≥50 were included, comparing GLP-1RA users (liraglutide, semaglutide, dulaglutide, exenatide, albiglutide) to non-users. Propensity-score matching balanced demographics and comorbidities. Incident dementia, defined by ICD-10 codes, was analyzed with Cox proportional-hazards models.

**Results:**

Matched cohorts (n = 147,505 each) had similar baseline characteristics. Dementia incidence was lower in GLP-1RA users (0.20% versus 0.44%), with a hazard ratio of 0.30 (95% CI 0.28–0.33; p < 0.001).

**Conclusions:**

GLP-1RA use was associated with a 70% reduced dementia risk, warranting further clinical evaluation.

## Introduction

Alzheimer's disease (AD) and related dementias (ADRD) are characterized by progressive cognitive decline and have emerged as significant global health challenges amid rapidly aging populations.^1^ It represents a significant global health challenge, increasing prevalence due to the aging population, which means the fifth leading cause of death among older U.S. residents.^2^ Despite recent FDA approvals of disease-modifying treatments for AD (e.g. aducanumab, lecanemab, and donanemab), concerns about their efficacy and safety persist.^3,4^ This uncertainty underscores the critical need for alternative strategies to decrease the ADRD risk. One promising approach is drug repurposing by exploring new uses for existing medications, which can expedite the development of novel treatments for ADRD.

Building on the growing interest in innovative therapies for type 2 diabetes (T2D), glucagon-like peptide-1 receptor agonists (GLP-1RAs) have emerged as promising agents not only for glycemic control but also for their cardiovascular, renal, and weight-related benefits.^5^ Beyond these established effects, emerging evidence suggests that GLP-1RAs may also influence the pathophysiology of ADRD by modulating neuroinflammatory pathways and reducing amyloid plaque accumulation.^6^ Population-based studies have hinted at a potential association between GLP-1RA use and a lower risk of ADRD.^7^ However, prior findings remain inconclusive due to limited sample sizes, heterogeneous populations, variable definitions of dementia, and inconsistent adjustment for confounding factors.

This study aims to address this gap in the literature by evaluating the association between GLP-1RA use and ADRD risk in a real-world population. We conducted a population-based cohort study using a target trial emulation approach, which simulates a randomized controlled trial by leveraging observational data. By doing so, we aim to provide more definitive evidence on the potential neuroprotective effects of GLP-1RAs, with implications for ADRD prevention and managing T2D.

## Methods

### Study design and data source

We performed a retrospective cohort analysis utilizing the TriNetX “Compare Outcomes” platform. TriNetX is a global federated research network comprising electronic health record (EHR) data from 142 healthcare organizations, including academic medical centers, community hospitals, and outpatient clinics primarily located in North America, with additional sites globally. The platform provides real-time longitudinal patient data on demographics, diagnoses, medication prescriptions, laboratory results, procedures, and clinical encounters.

### Ethical approval

The analysis was conducted on fully de-identified patient data. As such, it was exempt from institutional review board (IRB) review and informed consent requirements under the HIPAA Privacy Rule (Sections 164.514[a] and 164.514[b][1]). De-identification compliance was certified by a qualified expert, most recently reaffirmed in December 2020.

### Cohort definitions

We defined two patient cohorts:

- *GLP-1 receptor agonist (RA) users:* Patients prescribed any GLP-1 RA medication (liraglutide, semaglutide, dulaglutide, exenatide, or albiglutide). The first recorded GLP-1 RA prescription date defined the index date.
- *Non-GLP-1 RA users (Comparator group):* Patients without any documented exposure to GLP-1 RAs. The index date was defined as the date of a randomly selected qualifying outpatient clinical encounter.

### Inclusion and exclusion criteria

Eligible patients were adults aged ≥50 years with at least one year of available clinical data before their index date. Patients were excluded if they had a pre-existing dementia diagnosis (ICD-10 codes: G30., *F01*, F02.8*, F03.*) insufficient medical records, defined as fewer than two documented clinical encounters in the year prior to index or missing key baseline covariates (e.g. age, sex, BMI, or comorbidities) needed for propensity score matching.

### Outcome definition

The primary outcome was the incident diagnosis of dementia, defined by at least two dementia-related ICD-10 codes (including Alzheimer's disease [G30.*], dementia in other conditions [F02.80, F02.81], psychotic disorders secondary to medical conditions [F06.2], delirium [F05], and delusional disorders [F22]), recorded on separate clinical encounters. Outcome follow-up started the day after the index date and continued until dementia diagnosis, death, last available medical record, or end of study period.

### Covariates

Baseline covariates included:

- *Demographic factors:* age (modeled as a continuous variable), sex (binary: male/female), and body mass index (BMI, continuous).
- *Clinical comorbidities:* diabetes mellitus (ICD-10: E08–E13), hypertension (I10), hyperlipidemia (E78.5), ischemic heart disease (I20–I25), chronic kidney disease (N18), nicotine dependence (F17)
- *Concurrent medications:* insulin, metformin, sulfonylureas, and dipeptidyl peptidase-4 (DPP-4) inhibitors. Dosage information was not uniformly available across contributing healthcare organizations and thus was not included.

### Propensity score matching

We used propensity score matching (PSM) to control for baseline confounders. Propensity scores estimating the probability of GLP-1 RA exposure were derived using logistic regression that included all baseline covariates. GLP-1 RA users were matched 1:1 without replacement to non-users via nearest-neighbor matching with a caliper width of 0.1 standard deviations of the logit propensity score. Covariate balance post-matching was evaluated using standardized mean differences (SMDs), with SMD <0.1 indicating acceptable balance.

### Statistical analysis

We employed Cox proportional hazards regression models to calculate hazard ratios (HRs) and 95% confidence intervals (CIs) for dementia outcomes associated with GLP-1RA use. All models were applied to cohorts after propensity score matching, which controlled for baseline demographic, clinical, and medication-related covariates. The proportional hazards assumption was verified using Schoenfeld residual tests, and competing risks from mortality were addressed via Fine–Gray subdistribution hazard modeling. Statistical significance was set at a two-sided p-value <0.05. Analyses were performed using TriNetX analytics.

## Results

### Cohort characteristics

The initial cohorts included 635,396 GLP-1 RA users and 541,928 non-users. After propensity score matching, each group was reduced to 147,505 patients, achieving excellent covariate balance (Table 1). Matched cohorts had comparable demographics: mean age was approximately 60.6 years for GLP-1 users and 60.7 years for non-users, and females comprised 52.3% and 54.9% of each group, respectively (all SMD < 0.1). Clinical characteristics, including diabetes, hypertension, hyperlipidemia, chronic kidney disease (CKD), and nicotine dependence, were well balanced post-matching (all SMD < 0.1).

**Table 1.** Cohort characteristics after propensity matching.

Characteristics	GLP-1 Users (n = 147,505)	Non-GLP-1 Users (n = 147,505)	Standardized Mean Difference (SMD)
Age at Index (Mean ± SD)	60.6 ± 9.2	60.7 ± 10.1	0.008
Female (%)	52.3	54.9	0.052
Male (%)	47.6	45.1	0.051
Black or African American (%)	16.7	16.3	0.011
White (%)	71.4	71.4	<0.001
Overweight and Obesity (%)	40.3	41.7	0.029
Essential Hypertension (%)	63.8	62.7	0.024
Diabetes Mellitus (%)	50.8	47.0	0.076
Nicotine Dependence (%)	16.4	15.2	0.034
Hyperlipidemia (%)	44.5	43.6	0.018
Chronic Kidney Disease (%)	16.2	15.7	0.011
SGLT2 Inhibitors (%)	5.2	4.1	0.055
Metformin (%)	25.7	22.8	0.068
Sulfonylureas (%)	12.8	10.9	0.060
DPP-4 Inhibitors (%)	6.1	5.1	0.045

Characteristics of GLP-1 receptor agonist users and matched non-users after propensity score matching. Standardized mean differences (SMD) < 0.1 indicate acceptable covariate balance between groups.

### Incident dementia outcomes

Over median follow-up periods of 863 days (GLP-1 users) and 416 days (non-users), incident dementia occurred in 298 patients (0.20%) in the GLP-1 cohort and 655 patients (0.44%) in the non-user cohort. The unadjusted risk ratio demonstrated significantly lower dementia risk in GLP-1 RA users (RR = 0.46, 95% CI: 0.40–0.52; absolute risk difference: −0.24%, 95% CI: −0.30% to −0.18%; p < 0.001) (Table 2).

**Table 2.** Incident dementia outcome.

Group	Number of Patients	Incident Dementia Cases (%)	Median Follow-up (days)	Risk Ratio (95% CI)	Hazard Ratio (95% CI)	Absolute Risk Difference	p
GLP-1 Users	147,505	298 (0.20%)	863	0.46 (0.40–0.52)	0.30 (0.28–0.33)	−0.24%	<0.001
Non-GLP-1 Users	147,505	655 (0.44%)	416	Reference	Reference	Reference	Reference

Comparison of incident dementia cases between GLP-1 receptor agonist users and matched non-users, showing median follow-up durations, incident rates, risk ratios, hazard ratios, absolute risk differences, and p-values.

### Multivariable Cox proportional hazards analysis

Multivariable Cox regression analysis revealed that GLP-1 RA use was significantly associated with a reduced hazard of incident dementia (HR = 0.30, 95% CI: 0.28–0.33, p < 0.001). Additional factors associated with increased dementia risk included nicotine dependence (HR = 1.98, 95% CI: 1.84–2.13, p < 0.001) and CKD (HR = 1.32, 95% CI: 1.20–1.44, p < 0.001). Conversely, hyperlipidemia was associated with a decreased hazard (HR = 0.84, 95% CI: 0.78–0.91, p < 0.001). Age, sex, diabetes, and BMI were not significantly associated with dementia outcomes in adjusted models. These findings indicate a robust and clinically meaningful association between GLP-1 RA exposure and reduced dementia risk among older adults in this large, matched cohort (Table 3).

**Table 3.** Cox proportional hazards analysis of dementia risk: detailed cox proportional hazards analysis assessing dementia risk associated with individual GLP-1 receptor agonists and key covariates.

Covariate	Hazard Ratio (95% CI)	Coefficient	Standard Error	z	p
Liraglutide	0.30 (0.28–0.33)	−1.198	0.045	−26.507	<0.001
Semaglutide	0.30 (0.28–0.33)	−1.198	0.045	−26.507	<0.001
Dulaglutide	0.30 (0.28–0.33)	−1.198	0.045	−26.507	<0.001
Exenatide	0.30 (0.28–0.33)	−1.198	0.045	−26.507	<0.001
Albiglutide	0.30 (0.28–0.33)	−1.198	0.045	−26.507	<0.001
Male Sex	0.95 (0.89–1.01)	−0.054	0.034	−1.596	0.110
Age at Index	1.00 (1.00–1.01)	0.002	0.002	1.096	0.273
Nicotine Dependence	1.98 (1.84–2.13)	0.683	0.036	18.903	<0.001
Diabetes Mellitus	0.98 (0.89–1.08)	−0.024	0.049	−0.489	0.625
Hyperlipidemia	0.84 (0.78–0.91)	−0.171	0.039	−4.365	<0.001
Hypertension	1.09 (1.01–1.18)	0.086	0.039	2.185	0.029
Ischemic Heart Disease	0.91 (0.84–0.99)	−0.095	0.042	−2.239	0.025
Chronic Kidney Disease	1.32 (1.20–1.44)	0.274	0.046	5.928	<0.001
Insulin Use	1.15 (1.05–1.25)	0.136	0.043	3.189	0.001
DPP-4 Inhibitors	0.92 (0.80–1.06)	−0.082	0.070	−1.162	0.245
Metformin Use	1.01 (0.91–1.11)	0.005	0.052	0.104	0.917
Sulfonylureas	1.19 (1.06–1.33)	0.171	0.057	3.000	0.003

Hazard ratios (HR) are presented with 95% confidence intervals (CI), coefficients, standard errors, z-values, and p-values.

## Discussion

In this large, real-world cohort of over 295,000 patients, the use of GLP-1RAs was associated with a 70% reduction in the risk of dementia compared to non-users, based on robust statistical modeling using propensity score matching. Based on our matched cohorts of 147,505 patients, we observed 298 (0.20%) dementia cases in GLP-1RA users compared to 655 (0.44%) cases in the control group. The absolute risk difference of −0.24% indicates that GLP-1RA users had a lower absolute probability of developing dementia compared to non-users. Moreover, the multivariable Cox regression model, which accounts for potential confounding variables and comorbidities, confirmed a robust 70% reduction in dementia risk (HR = 0.30), emphasizing the independent protective effect of GLP-1RAs against dementia beyond other known risk factors.

Importantly, this 70% hazard ratio reduction (HR = 0.30) has profound clinical implications. Practically speaking, it implies that GLP-1RA use could significantly postpone the onset of dementia in older persons and high-risk groups, maintain cognitive function over extended periods of time, and lessen the burden that neurodegenerative illnesses place on society. Given the terrible effects dementia has on patients, families, and healthcare systems, even a small delay in onset could result in considerable gains in quality of life and economic savings.

Interestingly, and contrary to prior expectations, our analysis demonstrated that hyperlipidemia was associated with a reduced risk of dementia (HR = 0.80). This unexpected finding may be partially explained by potential biases, such as survival bias, where individuals with hyperlipidemia might receive more intensive medical care and monitoring, thereby reducing their dementia risk. Additionally, treatment with lipid-lowering therapies, particularly statins, which have been suggested to possess neuroprotective effects, could contribute to the observed association.^8^ Other risk factors, such as nicotine dependence (HR = 1.98) and chronic kidney disease (HR = 1.32), were associated with an increased risk of dementia. Conversely, age, sex, diabetes, and BMI percentile were not statistically significant after adjustment, reflecting their lack of influence on the risk of dementia and GLP-1RA use. These findings highlight the complex interplay between various comorbidities and dementia risk and underscore the importance of adjusting for confounding factors in observational studies.

Dementia, including AD and other subtypes, remains one of the leading causes of disability and mortality among older adults, imposing significant psychological and financial burdens on families and healthcare systems.^9^ The lack of a definitive cure further emphasizes the need to explore new therapeutic and preventive strategies. These findings highlight the significant neuroprotective potential of GLP-1RAs and support their application in a broader, real-world population beyond individuals with T2D.

Our study results are generally consistent with a systematic review and meta-analysis published in 2023, which suggested that GLP-1RAs might reduce the incidence of various types of dementia in patients with T2D (RR = 0.72).^10^ But our study is substantially stronger for several reasons. First, we drew from a heterogeneous, real-world cohort that included not only patients with T2D but also individuals with obesity and cardiovascular risk factors, whereas the meta-analysis was confined to diabetic trial populations. Second, our rigorous propensity score matching was adjusted for a comprehensive set of demographics, clinical, and socioeconomic confounders. In contrast, the meta-analysis pooled studies with variable adjustment approaches. Moreover, the meta-analysis exhibited high heterogeneity in study designs, patient characteristics, and dementia definitions, which may have attenuated its pooled effect estimate.

Similarly, our findings align with several observational studies,^10–12^ particularly a cohort study using OneFlorida + electronic health record data from the U.S., which reported a 33% lower incidence of dementia among T2D patients using GLP-1RAs compared to non-users. Nevertheless, these prior studies were geographically restricted and focused exclusively on T2D populations, raising concerns about the generalizability of the findings. Therefore, while our results contribute to the growing body of evidence suggesting a potential neuroprotective role of GLP-1RAs, they must be interpreted cautiously. Further robust, large-scale studies including broader patient populations, such as individuals with obesity or cardiovascular risk factors, are needed to validate these associations and better understand their applicability beyond T2D patients. Several preclinical studies have elucidated key molecular mechanisms by which GLP-1RAs confer neuroprotection: in animal models, these agents reduce pro-inflammatory cytokines (IL-1β, TNF-α), inhibit harmful A1 astrocyte formation and microglial activation, and decrease amyloid-β and tau aggregation while promoting neuronal progenitor proliferation and synaptic resilience.^13–15^ Translating these findings to humans, randomized trials such as ELAD and the EVOKE/EVOKE + programs have demonstrated that GLP-1RA treatment preserves cerebral glucose metabolism, enhances functional connectivity in memory-related brain networks on functional MRI, and slows progression of early AD pathology. Together, this bench-to-bedside evidence provides a biologically plausible foundation for our observed 70% reduction in dementia risk (HR = 0.30), indicating that the same anti-inflammatory and neurotrophic pathways active in preclinical models are likely mediating the robust clinical protection seen in our real-world cohort.^16,17^

Our findings complement and extend those of De Giorgi et al.,^18^ who conducted a large-scale propensity score–matched analysis within the TriNetX US Collaborative Network to evaluate 12-month neurological and psychiatric outcomes of semaglutide compared with other antidiabetic agents in patients with T2D. Their work demonstrated no increased risk of adverse neuropsychiatric events and suggested potential protective associations with cognitive deficit and nicotine misuse. While both studies leverage TriNetX data, the scope and objectives differ substantially. De Giorgi et al. focused solely on semaglutide and included cognitive outcomes as part of a broad neuropsychiatric safety profile, whereas our study examines the entire GLP-1RA class (liraglutide, semaglutide, dulaglutide, exenatide, albiglutide) with incident dementia as the primary outcome of interest. Moreover, our cohort draws from a global TriNetX dataset spanning 142 healthcare organizations across multiple continents, includes adults aged ≥50 years regardless of diabetes status, applies a longer median follow-up period (863 days in GLP-1RA users versus 416 days in non-users), and uses dementia-specific definitions requiring confirmation across two separate encounters. We also incorporate Fine–Gray competing risk models to account for mortality, an important factor in older populations. These differences expand the generalizability and dementia-specific relevance of our findings, building upon and extending the cognitive outcome signal first suggested by De Giorgi et al.^18^

### Strengths

This study utilized a rigorous matching technique on the TriNetX data network, which includes 143 healthcare organizations across multiple continents, thus overcoming the geographical variation among patients in previous studies. It also included patients with T2D and other users of GLP-1RAs, enabling the use of GLP-1RAs as a neuroprotective drug in a more inclusive, real-world population. Given that no currently approved medications modify the course of dementia and all available treatments remain symptomatic,^9^ the importance of working on prevention and halting progression in the preclinical stage becomes paramount. Therefore, it is worth reconsidering GLP-1RAs not only as antidiabetic or anti-obesity agents but also as a neuroprotective drug, particularly for the prevention of dementia in high-risk individuals.

### Limitations

Despite the strengths of this study, including its large sample size, global scope, and careful matching methodology, several limitations should be acknowledged. First, although propensity score matching helped minimize observable confounding, the observational design inherently limits causal inference. Randomization was not performed, and residual bias may persist. Additionally, important confounding factors such as socioeconomic status, cognitive reserve, educational level, and family history of dementia were not accounted for in the dataset.

Unmeasured confounding remains a key concern. Lifestyle behaviors (e.g. diet, physical activity), genetic predisposition, and access to healthcare were not available in the data and could influence both the likelihood of receiving GLP-1RA therapy and the risk of developing dementia. These unobserved factors may bias the observed associations.

Second, the study relied exclusively on diagnostic coding (ICD-10) for dementia identification. While this approach reflects real-world clinical practice and facilitates large-scale analysis, it is subject to potential misclassification. ICD-10 codes may not reliably differentiate between dementia subtypes and may fail to capture early or atypical cases. Furthermore, diagnostic accuracy may vary across institutions and healthcare systems, affecting consistency. Third, the relatively short follow-up period may limit the detection of all incident dementia cases, particularly given the long prodromal phase of many neurodegenerative disorders. Longer observation periods are essential to more fully understand the temporal relationship between GLP-1RA use and dementia onset. Fourth, the study did not include neuropsychological assessments, imaging data, or biomarker validation to confirm diagnoses, which could have improved diagnostic specificity and differentiation among dementia types. Additionally, our analysis relied on ICD-10 coding, which remains the standard across the TriNetX network, even though ICD-11 is now available in some settings. This reflects current healthcare infrastructure rather than a study design choice. For the Cox proportional hazards models, we note that the TriNetX platform includes automated verification of proportional hazards assumptions using Schoenfeld residuals, ensuring model validity, although supplementary graphical diagnostics (e.g. Kaplan–Meier curves or residual plots) were not available for inclusion. Finally, no subgroup analysis was conducted based on specific types or dosages of GLP-1 receptor agonists, which limits the ability to determine whether particular agents or dose ranges are more effective in reducing dementia risk. Subgroup analyses by individual GLP-1RA agents, dosage levels, or diabetes status were not performed because these details were not uniformly available across the TriNetX network. The heterogeneity of prescribing practices and incomplete capture of dosing information limited our ability to conduct reliable stratified analyses.

These limitations underscore the need for future prospective, randomized, and mechanistically guided studies to validate our findings and better understand the potential neuroprotective role of GLP-1RAs. Given these findings, it is important to consider the clinical implications for dementia prevention and treatment. We can exploit the neuroprotective effects of GLP-1 receptor agonists for older adults at high risk of dementia, including those with metabolic syndrome, T2D, obesity, or cardiovascular risk factors. Importantly, these benefits may extend beyond traditional glycemic control, suggesting a broader therapeutic role for GLP-1RAs in dementia prevention.

If validated in future randomized controlled trials, clinicians may start considering GLP-1RAs not only for metabolic regulation but also for cognitive protection in at-risk populations, even among non-diabetic individuals. This could mark a significant shift in current clinical practice, expanding the use of GLP-1RAs based on cognitive risk profiles rather than solely metabolic indications. However, before widespread clinical adoption, rigorous randomized controlled trials specifically targeting cognitive outcomes in non-diabetic populations are essential. These studies would need to confirm the causal relationship between GLP-1RA use and reduced dementia risk and identify optimal patient groups, treatment durations, and dosing strategies.

Broader implementation of GLP-1RAs as preventive therapy could significantly reduce the incidence of dementia, ultimately reducing the healthcare and caregiver burden associated with neurodegenerative diseases, and improving quality of life for aging populations globally.

### Conclusion

In conclusion, this cohort study, across many patients from different continents, demonstrated that GLP-1RAs are associated with a significantly reduced risk of incident dementia, not just in T2D patients but also in all users of this medication. This supports the use of GLP-1RAs as neuroprotective agents and their potential use as a dementia preventative. We recommend that future research focus on randomized controlled trials, a detailed study of the types of GLP-1RAs and optimal doses, the use of neuroimaging and biomarkers in diagnosis, and a longer patient follow-up period. If its effectiveness is confirmed, it will represent a significant advance and revolution in dementia prevention for patients at risk, transforming the approach to neurodegenerative disease prevention.
